# Clinical Efficacy of Probiotic Therapy on Bowel-Related Symptoms in Patients with Ulcerative Colitis during Endoscopic Remission: An Observational Study

**DOI:** 10.1155/2022/9872230

**Published:** 2022-01-17

**Authors:** Jin Lee, Su Bum Park, Hyung Wook Kim, Hong Sub Lee, Sam Ryong Jee, Jong Hun Lee, Tae Oh Kim

**Affiliations:** ^1^Division of Gastroenterology, Department of Internal Medicine, Haeundae Paik Hospital, Inje University College of Medicine, Busan 602-702, Republic of Korea; ^2^Division of Gastroenterology, Department of Internal Medicine, Pusan National University Yangsan Hospital, Yangsan 626-770, Republic of Korea; ^3^Division of Gastroenterology, Department of Internal Medicine, Inje University Busan Paik Hospital, Inje University College of Medicine, Busan 602-702, Republic of Korea; ^4^Division of Gastroenterology, Department of Internal Medicine, Dong-A University Medical Center, Busan, Republic of Korea

## Abstract

**Background:**

A substantial percentage of patients with ulcerative colitis (UC) have irritable bowel syndrome- (IBS-) like symptoms despite adequate treatment and endoscopic remission. In this study, we evaluated the clinical efficacy of probiotic therapy for residual IBS-like symptoms in patients with UC in endoscopic remission.

**Methods:**

We conducted a multicenter, observational study between April 2018 and December 2020 across two university hospitals in Korea. Patients with UC whose IBS-like symptoms persisted during endoscopic remission were included in this study. Endoscopic remission was defined as a Mayo endoscopic score ≤ 1, and IBS-like symptoms were defined as those meeting the ROME-IV diagnostic criteria. A Biotop capsule® (*Lactobacillus acidophilus*, 75 mg; *Clostridium butyricum* TO-A, 25 mg; *Bacillus mesentericus* TO-A, 25 mg; and *Streptococcus faecalis* T-110, 5 mg) was administered three times daily for one month. All patients completed bowel-related symptom questionnaires and short inflammatory bowel disease questionnaires (SIBDQs) at the start and end of the 4-week treatment period.

**Results:**

A total of 43 patients were enrolled and analyzed. Statistically significant improvements from baseline were observed at the end of the 4-week treatment. The total SIBDQ score improved from 50.6 to 53.6 (*P* = 0.005). SIBDQ scores of bowel function (*P* = 0.018), systemic function (*P* = 0.040), and social function (*P* = 0.005) improved. Stool frequency and Bristol stool scale scores improved after probiotic therapy (*P* < 0.05).

**Conclusion:**

This study showed that probiotic administration improved bowel-related symptoms and quality of life in patients with UC whose IBS-like symptoms persisted during endoscopic remission. As this is an observational study and has no placebo-controlled arm, further prospective randomized controlled trials are needed to confirm these results.

## 1. Introduction

Ulcerative colitis (UC) is a chronic inflammatory disease that causes localized inflammation in the mucosa or submucosa of the colon. UC causes symptoms such as abdominal pain, diarrhea, and hematochezia, which improve and worsen repeatedly throughout life. The treatment goals of UC are both clinical and endoscopic remission and maintenance of remission. Widely used and effective treatments for UC include 5-aminosalicylates (ASA), thiopurine, corticosteroids, and biologic agents.

However, a substantial percentage of patients with UC have bowel-related symptoms that persist despite adequate treatment and endoscopic remission [1–4]. This clinical phenomenon has been described as irritable bowel syndrome- (IBS-) like residual symptoms. Colombel et al. reported that endoscopic and histologic remission did not achieve complete remission of symptoms in patients with UC [5]. In another population-based cohort study, 30% of patients who achieved clinical and endoscopic remission using conventional medications complained of IBS-like symptoms [6]. IBS-like symptoms, including abdominal bloating and changes in the pattern or frequency of stool, seriously deteriorate the quality of life in patients with UC who have achieved disease remission. However, conventional treatment is generally ineffective in patients whose bowel-related symptoms persist during endoscopic remission [7]. Although the causes of both UC and IBS are unclear, impairment of the enteric nervous system, changes in intestinal flora, or activation of the brain-gut axis is considered to cause symptoms. In a meta-analysis of 21 studies [8], patients with IBS who were treated with probiotics had improved overall symptoms and quality of life, which was thought to be a result of the improvement of changes in the intestinal flora and incongruity of the enteric nervous system.

Previous studies have reported on the treatment of UC with probiotics [8–12], and there were remarkable results showing that probiotic therapy was not inferior to conventional medications such as 5-ASA [13]. However, the efficacy of probiotics in patients with UC whose bowel-related symptoms persist during endoscopic remission has not been well studied. In this study, we evaluated the clinical efficacy of probiotic therapy for bowel-related symptoms in patients with UC during endoscopic remission.

## 2. Materials and Methods

### 2.1. Patients

Between April 2018 and December 2020, we conducted a multicenter observational study across two university hospitals in Korea (CRIS Registration Number: KCT0002879). Patients with UC whose IBS-like symptoms persisted during endoscopic remission were included in this study. Endoscopic remission was defined as a Mayo endoscopic score (MES) ≤ 1, and IBS-like symptoms were defined as those meeting the ROME-IV diagnostic criteria. The following exclusion criteria were applied: patients < 18 years old; MES ≥ 2; proctitis or proctosigmoiditis due to other causes such as infection, medication, radiation, ischemia, or Crohn's disease; and inability to provide informed consent.

### 2.2. Study Protocol

This study was designed to evaluate the clinical efficacy of probiotic therapy in patients with UC whose IBS-like symptoms persisted during endoscopic remission. A Biotop capsule® (Daewoong Pharm, Seoul, Korea) was administered three times daily for one month. The capsule was a preparation containing *Lactobacillus acidophilus* (75 mg), *Clostridium butyricum* TO-A (25 mg), *Bacillus mesentericus* TO-A (25 mg), and *Streptococcus faecalis* T-110 (5 mg). All patients completed bowel-related symptom questionnaires and short inflammatory bowel disease questionnaires (SIBDQs) at the start and end of the 4-week study period.

### 2.3. Evaluation of Outcomes

The primary outcome was the daily frequency of stools and abdominal pain before and after probiotic treatment. The secondary outcomes were changes in stool form and quality of life. The outcomes were assessed using bowel-related symptom questionnaires and SIBDQs at the start and end of the 4-week study period. The SIBDQ is a 7-point scale with a total of 10 questions regarding intestinal symptoms (symptom), overall body function (behavior), emotional wellbeing (emotion), and social factors (social factors). For each question, the score ranges from 1 (“I have a very serious problem”) to 7 points (“I have no problem”). The total score ranges from a minimum of 10 points to a maximum of 70 points, and a high total score indicates a high quality of life. IBS-like symptoms before and after probiotic treatment were assessed by two questions: “How many times a day have you had abdominal bloating during the past 2 weeks?”; “How many times a day have you had loose bowel movements during the past 2 weeks?” The stool type was evaluated using the Bristol stool scale, which classifies stools into seven types: 1 and 2 indicate constipation; 3 and 4 are considered “normal” stools; and 5, 6, and 7 indicate diarrhea.

### 2.4. Statistical Analysis

Continuous data are presented as the mean ± standard deviation, and categorical data are presented as the number and percentage. Comparisons of paired variables were performed using a paired *t*-test, and normality was assessed using a Shapiro-Wilk test. For nonnormally distributed variables, a Wilcoxon signed-rank test was performed for comparison. Statistical significance was set at *P* < 0.05. All statistical data analyses were performed using SPSS 25.0 (IBM Corp., Armonk, NY, USA).

## 3. Results

### 3.1. Patient Characteristics

A total of 43 patients were enrolled and analyzed. The mean patient age was 44 years, and 19 patients (44%) were female, 42 (97%) were nonsmokers, and 14 (32%) consumed alcohol. We observed that 97% of patients received oral 5-ASA, 44% received 5-ASA suppositories, 11% received azathioprine, and 9% received anti-TNF. At the time of UC diagnosis, 21 patients (49%) had been diagnosed with proctitis, 12 (28%) had left-sided colitis, and 10 (23%) had extensive colitis. According to the ROME-IV criteria, 15 patients were classified as IBS with predominant diarrhea (IBS-D) and 28 patients were classified as IBS with mixed bowel habits. Of the total 43 patients, 32 (74%) had abdominal bloating symptoms.  Table 1 summarizes the baseline characteristics of the study participants.

### 3.2. Outcomes

After one month of treatment, the daily stool frequency in the last two weeks of treatment significantly improved (3.2 stools/day vs. 2.8 stools/day, *P* = 0.012), but there was no significant difference in the frequency of abdominal pain before and after treatment (1.1 vs. 0.9, *P* = 0.099). The Bristol scale significantly improved from 4.9 to 4.3 (*P* < 0.001). The changes in bowel-related symptoms before and after probiotic therapy are shown in Table 2.

**Figure 1:**
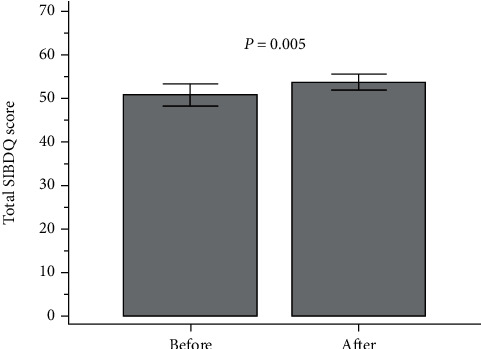
Total SIBDQ scores before and after treatment.

There was a significant improvement in the quality of life at the end of the 4-week treatment compared to baseline.  Figure 1 shows the changes in the total SIBDQ scores. The total SIBDQ score improved from 50.6 to 53.6 (*P* = 0.005). The following SIBDQ subscores improved: bowel function (15.0 to 15.9, *P* = 0.017), systemic function (9.7 to 10.3, *P* = 0.040), and social function (11.0 to 11.8, *P* = 0.005). There were no significant differences in emotional function before and after treatment.  Table 3 summarizes the changes in the SIBDQ scores before and after treatment. No adverse events occurred in any patient.

## 4. Discussion

IBS-like symptoms are reported to be frequent in patients with inflammatory bowel disease (IBD) with ongoing inflammation. A meta-analysis reported a substantial prevalence of IBS in patients with IBD compared with non-IBD controls [14]. Another recent meta-analysis reported similar results [15]. The causes of both IBS and UC remain unclear but are thought to be multifactorial: (1) subclinical mucosal inflammation, (2) visceral hypersensitivity, (3) genetic factors, (4) environmental factors, and (5) altered gut microbiome. This suggests that the pathophysiological mechanisms may overlap between the two disorders, and thus, IBS-like symptoms generally improve when UC remission is achieved [16].

However, a substantial percentage of patients with UC have bowel-related symptoms that persist despite adequate treatment and endoscopic remission [1–4]. This clinical phenomenon has been described as IBS-like residual symptoms, and it has been reported that occult inflammation may be related. Occult inflammation is an important pathophysiological explanation for residual bowel-related symptoms in patients with IBD in endoscopic remission [17]. A study assessing patients with IBD in clinical remission showed that IBS-like symptoms correlated with increased calprotectin levels, suggesting that occult inflammation may cause these symptoms [3]. IBS-like symptoms deteriorate the quality of life of patients with UC who are in remission. Conventional treatment is generally ineffective in patients with IBS-like symptoms persisting during endoscopic remission. Although previous studies have reported the effect of probiotics for the treatment of UC, the efficacy of probiotics for residual IBS-like symptoms in patients with UC in endoscopic remission has not been well studied. Our findings show that probiotic therapy may be a beneficial treatment option for these patients.

IBS is a functional disorder that causes gastrointestinal symptoms, including abdominal pain, bloating, constipation, and diarrhea. Although the prevalence of IBS varies according to country, a recent meta-analysis reported a global prevalence of 11.2% [18]. Because altered gut flora is considered a major etiology of IBS, many studies using probiotics in patients with IBS have been reported [19–22]. Although these studies report conflicting findings regarding the efficacy of probiotics, probiotics are considered a beneficial treatment option for patients with IBS. The gut microbiota plays an important role in UC and IBS, as it is known to modulate UC disease activity. Thus, the benefit of probiotics as treatment has been reported in several previous studies in patients with UC. *Escherichia coli* Nissle 1917 was first reported to be beneficial in preventing the recurrence of disease activity in quiescent UC compared with mesalazine [9]. Unfortunately, none of the previous studies addressed the use of probiotics in the treatment of IBS-like symptoms in patients with UC in endoscopic remission. Although the benefits of probiotics are still uncertain compared with placebo or mesalamine in UC with ongoing inflammation [23], probiotic use may be an attractive option in patients with UC with IBS-like symptoms.

This is the first study to report that probiotic therapy may be a beneficial treatment option for residual IBS-like symptoms in patients with UC in endoscopic remission. Our study showed that probiotics (Biotop capsule®) were effective in improving stool frequency and quality of life. Daily stool frequency significantly improved after the use of probiotics (3.2% vs. 2.8%, *P* = 0.012). The Bristol scale also showed statistically significant improvement. The total SIBDQ score improved from 50.6 points to 53.6 points and subscores of SIBDQ improved as follows: bowel function (*P* = 0.018), systemic function (*P* = 0.040), and social function (*P* = 0.005).

Our study has a few limitations. First, this study was an observational study, and researchers and enrolled patients were aware that all patients were receiving active treatment. The lack of comparison with a placebo control group limits inferences regarding the efficacy of probiotics. Second, due to the low prevalence of quiescent UC with residual IBS-like symptoms, the sample size was relatively small. Thus, the results of this study need further confirmation through large-scale randomized trials. Third, although the statistically significant differences were found for the SIBDQ scores and Bristol scale scores, there were only 3 points of differences for the SIBDQ and 0.6 points for the Bristol scale. Because the difference between the scores before and after treatment is small, there may be limitations in expecting the effect of treatment in actual clinical practice.

In conclusion, this study demonstrated that probiotics improved IBS-like residual symptoms, such as stool frequency, in patients with UC whose IBS-like symptoms persist during endoscopic remission. Furthermore, the administration of probiotics resulted in improvements in the quality of life and Bristol stool scale scores. This study results suggest that probiotic therapy may be an effective treatment option for residual IBS-like symptoms in patients with UC in endoscopic remission. As the sample size was relatively small and this study had no placebo-controlled arm, further placebo-controlled, double-blind randomized clinical trials are needed to confirm our results.

## Figures and Tables

**Table 1:** Baseline patient characteristics.

Variable	Overall (*n* = 43)
Age (years)	44.4 ± 12.5
Gender	
Female	19 (44.2)
Male	24 (55.8)
Presence of comorbidities	
Yes	8 (18.6)
No	35 (81.4)
Alcohol	
Yes	14 (32.6)
No	29 (67.4)
Smoking	
Current	1 (2.3)
Former, never	42 (97.7)
Disease duration in years	
<1 year	4 (9.3)
1–5 years	23 (53.5)
≥5 years	16 (37.2)
Disease extent at diagnosis	
Proctitis	21 (48.8)
Left-sided colitis	12 (27.9)
Extensive colitis	10 (23.3)
Oral 5-ASA	
Yes	42 (97.7)
No	1 (2.3)
5-ASA suppository	
Yes	19 (44.2)
No	24 (55.8)
Anti-TNF	
Yes	4 (9.3)
No	39 (90.7)
Azathioprine use	
Yes	5 (11.6)
No	38 (88.4)
IBS subtype	
IBS-diarrhea	15 (34.8)
IBS-mixed bowel habits	28 (65.2)
Symptoms of abdominal bloating	
Yes	32 (74)
No	11 (26)

Values are means ± standard deviation or number (%). 5-ASA: 5-aminosalicylic acid; TNF: tumor necrosis factor; IBS: irritable bowel syndrome.

**Table 2:** IBS-like symptoms before and after probiotic treatment.

	Before	After	*P* value
Number of abdominal bloating (per day in the last 2 weeks)	1.1 ± 1.6	0.9 ± 1.5	0.099
Stool frequency (per day in the last 2 weeks)	3.3 ± 1.4	2.9 ± 1.3	**0.012**
Bristol stool scale	4.9 ± 0.9	4.3 ± 0.7	**<0.001**

Values are means ± standard deviation or number (%). Bold style indicates statistical significance.

**Table 3:** SIBDQ scores before and after probiotic treatment.

	Before	After	*P* value
Bowel	15.1 ± 2.7	16.0 ± 2.1	**0.018**
Systemic	9.8 ± 1.8	10.3 ± 1.7	**0.040**
Emotional	14.7 ± 3.1	15.5 ± 2.8	0.071
Social	11.0 ± 2.3	11.8 ± 1.6	**0.005**
Total scores	50.6 ± 8.3	53.6 ± 6.5	**0.005**

Values are means ± standard deviation or number (%). SIBDQ: short inflammatory bowel disease questionnaire. Bold style indicates statistical significance.

## Data Availability

Data are available on request through the authors themselves.
